# The dual role of PORC in chloroplast thermotolerance

**DOI:** 10.1093/plphys/kiag227

**Published:** 2026-04-21

**Authors:** Eva Maria Gómez-Álvarez

**Affiliations:** Assistant Features Editor, Plant Physiology, American Society of Plant Biologists; Instituto de Biología Molecular y Celular de Plantas (CSIC-Universitat Politècnica de València), València 46022, Spain

The Global Annual to Decadal Climate Update predicted that the planet was going to breach the 1.5 °C limit in 2027 (Matthews and Wynes 2022); however, it has been observed that this limit was exceeded in 2025 (Hansen et al. 2025). Plants, which are bound to the surrounding climatic fluctuation, need to face the challenge of maintaining photosynthesis under thermal stress. While many studies explain how the cell nucleus and cytosol respond to heat (Evrard et al. 2013; Babbar et al. 2021; Réthoré et al. 2024), the mechanisms of the chloroplast response to heat are still being understood. It is known that heat stress can compromise membrane integrity and disrupt the protein homeostasis within the chloroplast (Hu et al. 2020). In a recent study, Alquraish and colleagues (2026) show that the chlorophyll biosynthesis enzyme PROTOCHLOROPHYLLIDE OXIDOREDUCTASE C (PORC) plays a role helping form protective areas called chloroplast stress granules (cpSGs) that protect the photosynthetic machinery during heat waves.

To understand this discovery, one must first consider the nature of the granules, which are dynamic compartments without membrane, that allow cells to organize their internal space. Stress granules represent a specific class of these condensates that are normally composed of proteins and messenger RNAs that aggregate when a cell is under stress (Protter et al. 2016). The enzyme PORC is central to this story because it catalyzes the light-dependent reduction of protochlorophyllide to chlorophyllide, an important step in the production of chlorophyll (Vedalankar et al. 2024). While PORC was previously known only for its metabolic role, its ability to be involved in the formation of cpSGs was unknown.

Alquraish et al., (2025) discovered that under normal conditions, PORC can be found everywhere throughout the chloroplast to facilitate chlorophyll production, but when *Arabidopsis thaliana* is exposed to acute 42 °C or prolonged 35 °C heat, the protein suffers a dramatic shift, condensing into these distinct, membrane-less cpSG aggrupation. This process is remarkably dynamic because the granules disassemble within 60 min once the heat disappears, allowing PORC to return to its diffuse state without being degraded. Furthermore, the formation of these granules is translation dependent, as shown by experiments with lincomycin, and appears specific to heat stress since other stressors like salt or drought do not trigger the same condensates.

To understand the internal environment of these granules, the researchers used proteomic profiling to identify the proteins that join PORC. They discovered a selective enrichment of PSI and PSII components, proteases such as the FtsH family, and chaperones like ROC4, which are all critical for repairing or stabilizing the photosynthetic machinery. The authors propose that cpSGs act as specialized microenvironments that protect these proteins from damage, preventing them from unfolding until the environment becomes favourable again (Fig. 1).

**Figure 1:**
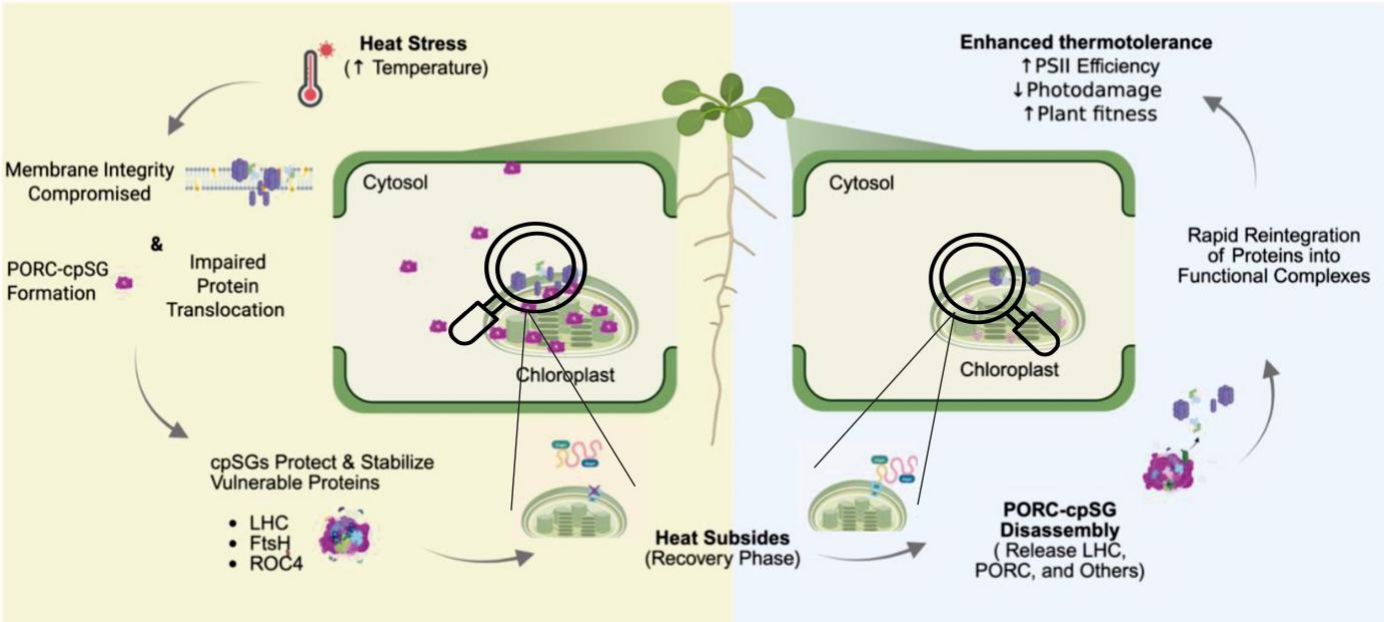
Model of the PORC contribution to chloroplast adaptation to heat stress. In the left panel, heat stress damages the membrane, impairing protein translocation and triggering spSG formation. In the right panel it is described how when heat subsides, the proteins come back to their normal functional complexes, allowing termotolerance. This system allows photodamage and improves plant PSII efficiency. Magnifying lenses allow us to understand what happens in the chloroplast. Adapted from Alquraish et al., 2026.

The study provides strong evidence for the importance of PORC in agricultural resilience, as PORC-overexpressing lines exhibited significantly better recovery, higher PSII efficiency, and improved growth rates after heat exposure. In addition, genetic disruption of PORC resulted in plants that were hypersensitive to heat, exhibiting stunted development and reduced survival. Interestingly, the team also found that heat disrupts the transport of nuclear-encoded proteins into the chloroplast, causing some PORC to aggregate in the cytosol. This work moves the field beyond simply observing condensates to proving their role in plant survival, suggesting that leveraging proteins like PORC could be a vital strategy for engineering heat-tolerant crops.

## Data Availability

No new data were generated or analyzed in support of this research.
